# The Mini Mental State Examination at the Time of Alzheimer's Disease and Related Disorders Diagnosis, According to Age, Education, Gender and Place of Residence: A Cross-Sectional Study among the French National Alzheimer Database

**DOI:** 10.1371/journal.pone.0103630

**Published:** 2014-08-05

**Authors:** Christian Pradier, Charlotte Sakarovitch, Franck Le Duff, Richard Layese, Asya Metelkina, Sabine Anthony, Karim Tifratene, Philippe Robert

**Affiliations:** 1 Department of Public Health, Nice University Hospital and University of Nice Sophia Antipolis, Nice, France; 2 Department of Clinical Research, Nice University Hospital Nice, Nice, France; 3 CoBTeK Research Unit, University of Nice Sophia Antipolis, Nice, France; 4 Memory Center – CMRR, Nice University Hospital, Nice, France; University Of São Paulo, Brazil

## Abstract

**The aim of this study:**

was firstly to describe the MMSE (Mini-Mental State Examination) score upon initial diagnosis of Alzheimer's disease and related disorders among the French population, according to age. Secondly, education, gender and place of residence were studied as factors potentially associated with delayed Alzheimer's disease diagnosis.

**Design:**

we conducted a cross sectional analysis of the French National Alzheimer database (BNA). Data from 2008 to 2012 were extracted. Patients were selected at the moment of their first diagnosis of AD (n = 39,451).

**Results:**

The MMSE score at initial diagnosis dropped significantly with increasing age. The test score increased with the degree of educational background regardless of age. Gender and place of residence were significantly related to the MMSE score, women and persons living in medical institutions having lower MMSE scores under the age of 90 years and at all educational levels.

**Conclusions:**

Health care professionals should be aware of these risk factors in order to maximize chances of earliest possible diagnosis of Alzheimer's disease and related disorders.

## Introduction

According to current estimates, there are over 24 million cases of Alzheimer's Disease worldwide and the condition has thus become a public health priority. In 2008, the French government launched the Third National Plan for “Alzheimer's disease and related disorders” (“Plan Alzheimer 2008–2012”). This plan was intended to develop research on Alzheimer's disease and related topics, facilitate early diagnosis and improve patient care, as well as provide support to families and helpers.

To improve early diagnosis and define optimal treatment strategies, cognitive screening is recommended. The Mini-Mental State Examination (MMSE), as developed by Folstein, Folstein, and McHugh (1975), is the most widely used among cognitive screening tools [1], [2], [3]. It is effective as a screening instrument to distinguish patients with cognitive impairment from those without. In practice, MMSE is used daily by clinicians to assess the progression and severity of the disease. The MMSE is included in numerous recommendations such as those put forward by the National Institute for Health and Clinical Excellence in the UK or the Haute Autorité de Santé in France.

The MMSE score is described in the literature as depending on patient age and educational level [4]. Thus, comparison of crude MMSE scores can be biased. However, when adjusting for these related factors, a lower MMSE score at the time of initial Alzheimer's disease diagnosis in subjects of similar age and educational level might reveal late detection leading to delayed treatment. Despite wide use of the MMSE score, no previous study has been performed examining the relation between MMSE score and age on a large sample of patients with Alzheimer's disease.

The aims of this study were therefore, first of all, to describe the MMSE score among the French population upon initial diagnosis of Alzheimer's disease and related disorders according to age. Secondly, factors associated with late Alzheimer's disease and related disorders diagnosis were studied. The French National Alzheimer database (BNA) [5], created within the Third National Plan for “Alzheimer's disease and related disorders” provided us with the opportunity to perform this study among a large sample of patients.

## Methods

### The French National Alzheimer Database (Banque Nationale Alzheimer: BNA)

The French National Database (known as “BNA”) is part of the French National Plan 2008–2012 for Alzheimer's disease and related disorders. Activity and epidemiological follow up data are recorded nationwide within specialized memory centres. The national network currently includes 400 “memory units” (CMs Consultation Memoire) and 28 “Memory Resources and Research Centres” (CMRRs Centres Memoire, de Ressources et de Recherche) and volunteer private specialists (neurologists, geriatricians and psychiatrists). The French National Alzheimer database has been described in a previous article [5]. Online information concerning the number of participating centres and patients is available at www.banque-nationale-alzheimer.fr. For each patient's visit, the BNA records a large number of variables including the MMSE score, the date of the first consultation in a memory centre, the diagnosis and the date of diagnosis. The BNA is supported by the French Ministry of Health. Records have been done under the conditions of the Commission Nationale de l'Informatique et des Libertés (CNIL), responsible in France for data protection and use with respect to the human identity and human rights.

### Selection of the study population

In this cross-sectional study, patients aged between 60 and 100 years were selected at the time of their first diagnosis of Alzheimer's disease or related disorders. Only one record was selected for each patient. The selected record was the first within the year following the initial consultation in the memory centre, in order to avoid including patients recorded in the BNA with a prior diagnosis of Alzheimer's disease or related disorders and thereby selecting only patients with a newly established diagnosis. Patients with missing data for MMSE score, education, age, gender, or place of residence were excluded.

### Selection of the studied variables

The full list of items, with the possible choices for each one, and a glossary of definitions can be accessed on the CMRR-Nice website (http://www.cmrr-nice.fr/) or the National Alzheimer Plan 2008–2012 website (http://www.plan-alzheimer.gouv.fr/mesure-no34.html). The CIMA (mandatory minimal information) currently displays 31 variables to be completed. For each procedure and each patient, this minimum data set must be transmitted to the BNA. The diagnosis procedure drawn up by the National Federation of CMRRs uses the ICD 10 classification. For this study, the following patient characteristics were extracted from the BNA: 1/age at the time of consultation; 2/education level categorized as follows: “primary school or none (ages 3–11)”, “secondary school - cycle 1” (ages 11–14), “secondary school - cycle 2” (ages 14–18) and “university level” (age >18); 3/place of residence grouped in three categories: “home alone”, “home with family” and “nursing home” (“EHPAD”) Accommodation for Elderly Dependent Persons or Elderly Care Institutions).

### Statistical Analysis

We modeled the MMSE score using multivariate linear regression. In order to ensure a Gaussian distribution, we transformed the MMSE score into the square root of the number of errors as already described by Jacquemin-Gadda *et al* [6]. We used fractional polynomials [7] with polynomials with one and two degrees of freedom to determine the form of the dependence of this transformed MMSE score on age. Our analysis showed that patient age squared best explained the MMSE score. For dependence on age alone, our model for this regression was 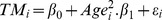
(1)

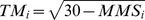
where MMS_i_ was the MMSE score of a patient i, Age_i_ was the age of this patient and ε_i_ was a centred normally distributed error with variance σ^2^. Then, we modeled the MMSE score as a function of age adjusted on educational level by 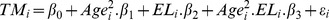
(2)where EL_i_ was a line-vector indicating the patient's educational level. Graphical examination of residuals of this regression indicated that the hypothesis of normality and homoskedasticity of errors was acceptable. We obtained the graph of the MMSE score depending on age, and depending on age adjusted on education, using the expectation of the MMSE score computed by the formula:
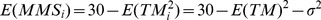
(3)


The influence of the other variables (gender and place of residence) was studied using the same type of model. We introduced one variable at a time in the regression model and its interactions with age squared and educational level. The expectation of the MMSE score was graphically represented as a function of patient age adjusted on education and on a covariate of our choice using formula (3). In order to check that the associations remained significant after adjustment, we estimated the complete model with all covariates significantly related to the MMSE score.

## Results

### The study population

**Figure 1:**
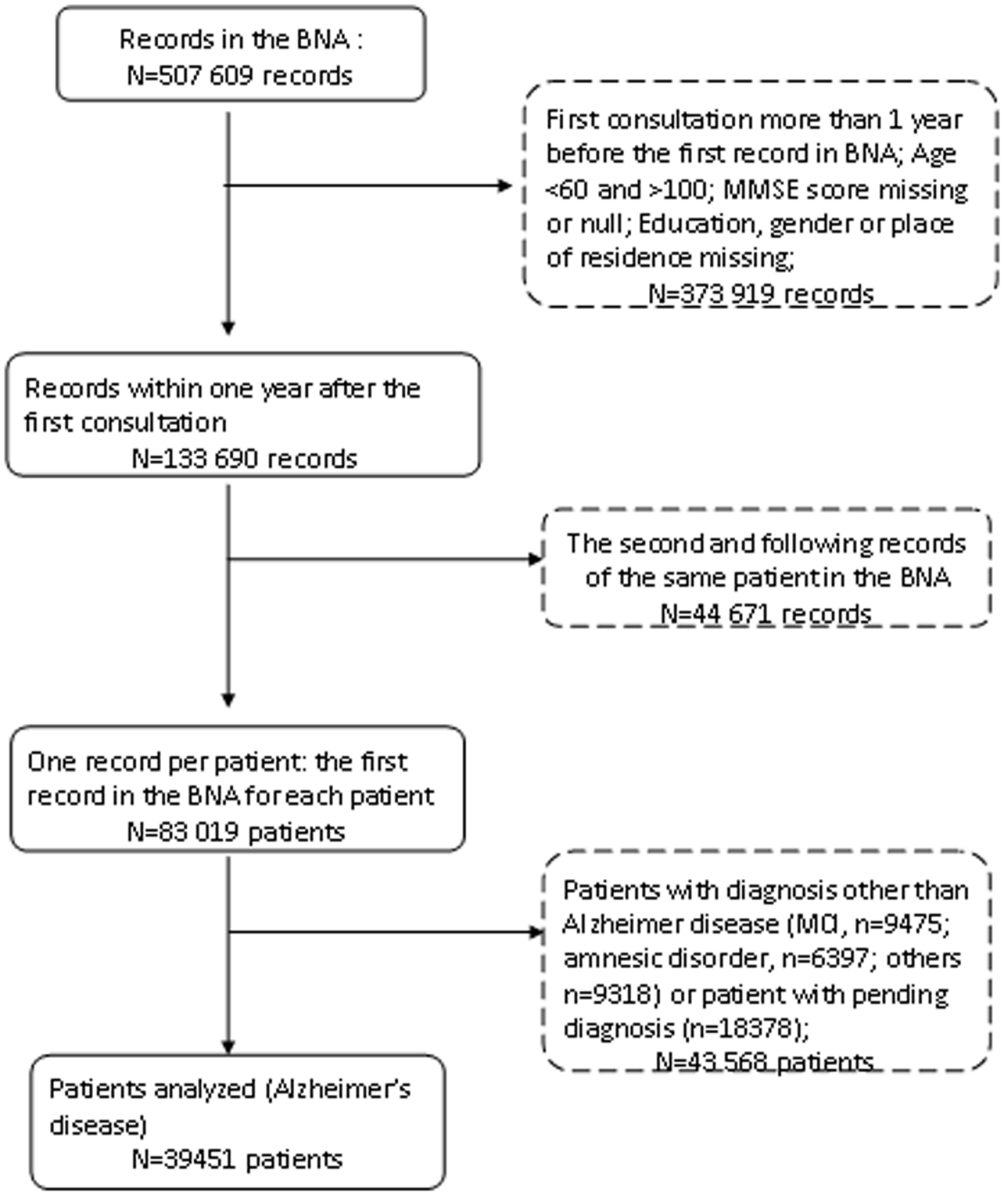
Selection of the patients included in the study.

From 507,609 records in the BNA, 133,690 records concerning 83,019 patients aged 60 to 100 years met our criterion to contain both their MMSE score and educational level. Among these patient records, 39,451 patients were diagnosed with Alzheimer's disease and related disorders, 9,475 patients with Mild Cognitive Impairment diagnosis, 9,318 patients had related pathologies or other diagnoses, while diagnosis was still pending for 18,370. Figure 1 illustrates the sample selection flowchart.

**Figure 2:**
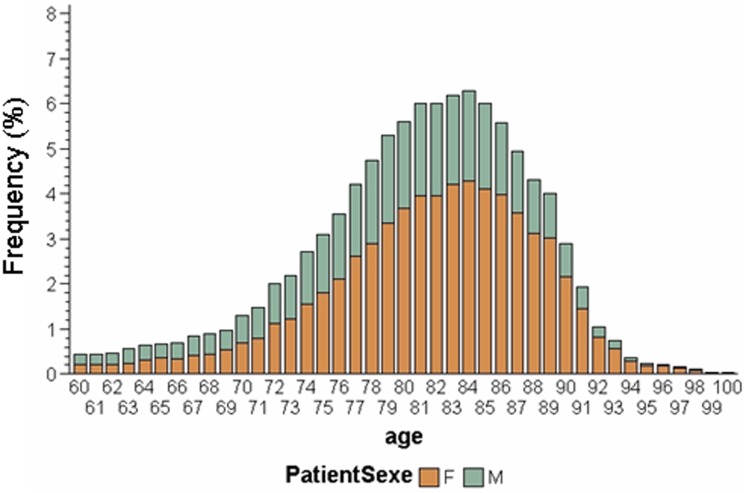
Distribution of men and women in the BNA sample according to age.

The mean age of our selected population with Alzheimer's disease and related disorders (n = 39,451) was estimated as 80.92±6.83. The mean MMSE score for this population was 18.91±5.48. The self-declared educational level was primary school for 57.5% of patients while 10.0% had no education. Most patients (83.4%) lived at home, either alone (8.6%) or with their family. Women contributed 65.4% of the sample and the proportion of women in the sample varied with the age (Figure 2): 50% for those aged 60–69 years, 59.4% for 70–79 years, 69.0% for 80–89 years.

### MMSE as a function of the age

**Figure 3:**
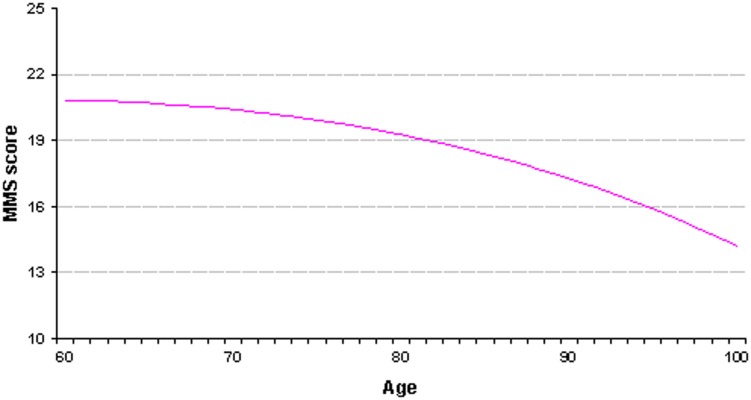
Patients with Alzheimer's disease and related disorders: the MMSE score at the time of first diagnosis according to age.

Figure 3 shows the curve obtained with the regression model (1), giving the expected MMSE score at the time of the first diagnosis of Alzheimer's disease and related disorders according to patient age. Age appeared strongly associated with the MMSE score (p<0.0001).

### MMSE as a function of age and educational level

**Figure 4:**
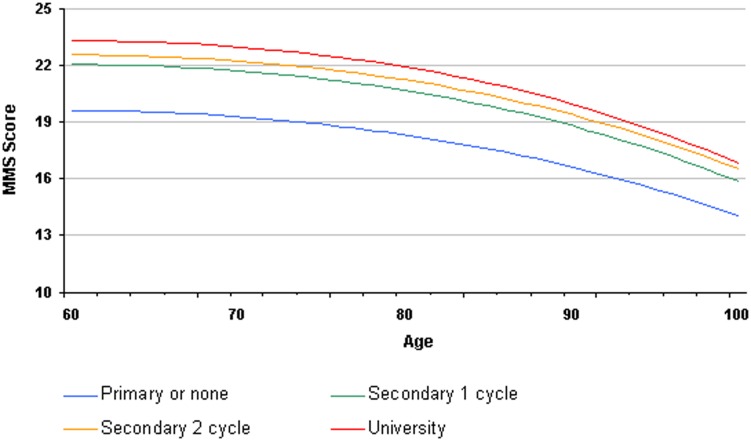
Patient with Alzheimer's disease and related disorders: MMSE score at the time of first diagnosis according to age and educational level.

In model (2), both age and educational level were strongly associated with the MMSE score (p<0.0001) as well as the interaction term (p = 0.0006). Within the same age group at first diagnosis, patients with primary or no education had systematically inferior MMSE scores compared to those of patients with secondary or university education (Figure 4). Despite the statistically significant interaction term, the distance between the four MMSE curves according to educational level remains quite stable throughout age groups with MMSE score more than 2 points lower at any age in the lowest educational level category compared to the secondary - cycle 1 category. Within the 3 highest educational levels there was about 1 point difference between the two extreme levels, the middle education level remaining between those two curves throughout all ages.

### Effect of gender

**Figure 5:**
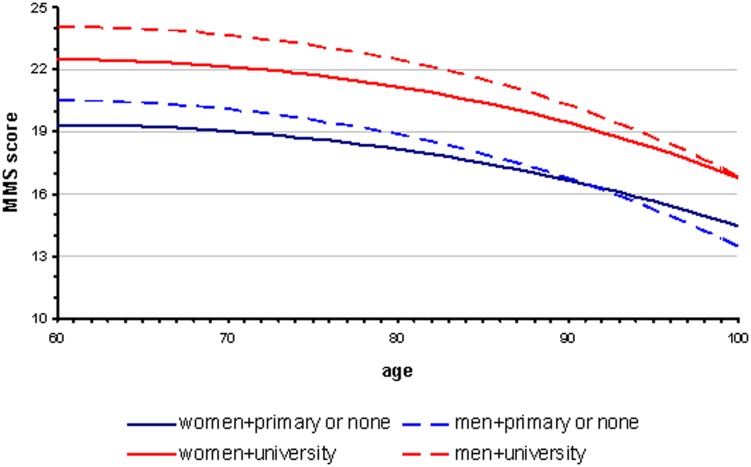
Patients with Alzheimer's disease and related disorders: MMSE score at the time of first diagnosis according to age, educational level and gender.

Gender was significantly associated with MMSE score adjusted on age and education (p<0.0001). Given the importance of the effect of education on the MMSE score, we took into account the interaction between education and gender which was statically significant (p = 0.0005). Figure 5 illustrates the effect of gender and education on the dependence of MMSE score on age. To clarify Figure 5, we kept only the extreme educational levels (“primary or none” and “university”). Among patients below 90 years of age, women consistently had a lower MMSE score than men of the same age and educational level.

### Effect of place of residence

Place of residence was significantly associated with MMSE score adjusted on age and education (p<0.0001). We likewise studied the interaction between place of residence and education, but this interaction was not significant (p = 0,74).

**Figure 6:**
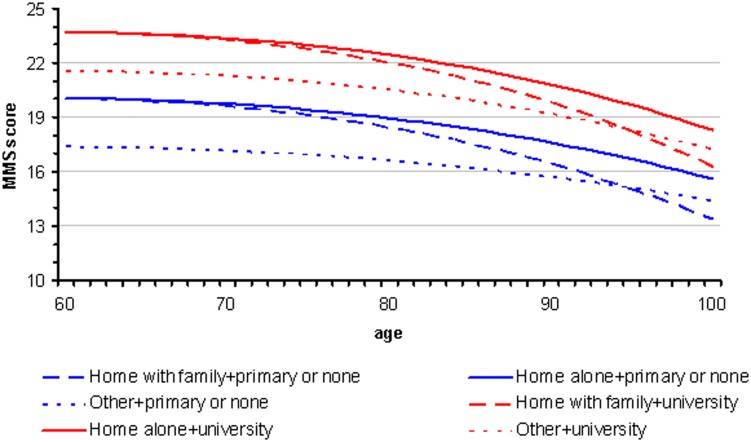
Patients with Alzheimer's disease and related disorders: MMSE score at the time of first diagnosis according to age, educational level and place of living.

Figure 6 shows that, regardless of education level and age at the time of diagnosis, patients living in institutions had systematically lower MMSE scores than patients living at home, whether alone or with their family.

## Discussion

This study, performed on a large number of French patients with Alzheimer's disease and related disorders whose diagnosis had been mainly established in Memory Units, suggests that not only age and education but also gender and place of residence were strongly associated with the MMSE score at the time of the initial diagnosis.

Our study showed that the MMSE score upon initial diagnosis depends non-linearly on patient age and decreases with age. This result is expected since age is the most well recognized risk factor for dementia. Population based studies have already shown a strong relationship between age and MMSE scores in the general population [8], [9]. This effect can be partly explained by cognitive decline related to aging. However, Alzheimer's disease with onset after the age of 80 years tends to have a slower course than earlier onset cases. Consequently, the transition from ‘normal ageing’ to ‘dementia’ may be less clear cut in the former and lead to a later presentation to clinical services (and lower MMSE at the time of diagnosis). Unfortunately, since we do not have any information in our database about the likely duration of symptoms or the severity of functional impairment at the time of diagnosis, it was not possible to adjust our analyses for these factors. It could not be excluded that if the analyses were adjusted for the severity of functional impairment, the score lines would have been parallel to the age axis.

Our study confirmed the strong correlation between MMSE score and education. We established that low educational level and older age limited cognitive performance during the MMSE. The fact that the MMSE score depends on these two factors was shown in studies of the native American population in 1991 by Ganguli et al and in 1993 by Crum et al [4], [9]. More recently, studies concerning the Hispanic population in the USA led to similar conclusions [10].

The study also suggests that women are diagnosed at more advanced stages of cognitive decline than men below 90 years. This result might be explained by selective survival of men with lower propensity to dementia, as reported by Chene et al [11]. Moreover, women are living longer than men. We observed this in our sample, where the proportion of women increases with age (Figure 2). Therefore, women are more likely to be diagnosed at a more advanced stage of the disease than men. However, our analysis was adjusted on age so it is unlikely that this fact alone could explain the observed phenomenon.

Delay in diagnosis for women may also be partly due to widowhood (more frequent among old females than males) and consequent absence of a career who might bring them to medical attention sooner rather than later.

On the other hand, earlier diagnosis of MCI in men compared to women has been already reported [12] as well as a different rate of progression of Alzheimer's disease according to gender [13], [14]. A possible explanation is the biological difference between women and men as some studies suggest that women have a smaller brain reserve [15], [16], [17]. Moreover, animal experimental data seem to support the presence of gender differences in pathology and memory decline, females being affected earlier and to a higher degree than males [18], [19], [20]. Nevertheless, we cannot rule out the role of confounders such as social life or profession. It is also possible that these confounders play an important role in the awareness of patients and their families of the cognitive disorders, and in the approach by specialists of these cognitive impairments that appear different between men and women.

MMSE scores of patients living in medical institutions for the elderly (n = 4357) upon initial diagnosis of Alzheimer's disease and related disorders were consistently lower than those of patients living at home. It is likely that a significant number of patients with Alzheimer's disease and related disorders in these institutions are not diagnosed or are diagnosed late. This may be due to the fact that patients living in these institutions are less independent and their cognitive decline has less impact on their daily living so that they are unaware of it. Alternately, it may be that care-givers are less attentive to these patients, attributing their condition to age. Further work is required to study this issue.

A surprising effect is that for very old patients (over 80 years), the MMSE score for those living at home on their own decreased at a much slower rate and became systematically superior to the score of patients living at home with their family. This could be because patients living on their own can do so thanks to their good cognitive abilities, or because those patients maintain their cognitive abilities thanks to the stimulus provided by the requirements necessary for independent living.

We performed this study on a large number of patients and used multivariate nonlinear regression which is more flexible than standard linear regression. Additional analysis was conducted in order to compare our results with those obtained using non-parametric regression for cognition in the proportional-odds model with thresholds as described by Jacquemin-Gadda *et al.* [21], [22]. With this more sophisticated model results were very close to those obtained with nonlinear multivariate regression, so that this simpler model was chosen as its performance was satisfactory (no more than 5–10% of error between the two models).

This study also has some limitations. First, the BNA is not, to date, a comprehensive dataset of all Alzheimer's disease patients in France and questions remain concerning the representativeness of the study. Moreover, some patients have not been included due to missing values on the inclusion criteria (e.g. diagnosis or date of first consultation). This could have biased the representativeness of our sample. A difference in age or gender between these not included patients and our selected population would therefore not be surprising. Finally, as expected, there was less patients over the very old ages (>90 years). Thus, our estimates could be less precise for the very old patients.

In conclusion, this study using the French National Alzheimer database indicated that, at the time of diagnosis, the level of cognitive performance in patients with a diagnosis of Alzheimer's disease and related disorders not only depends on age and education, but also on other factors such as gender and place of residence. Health care professionals should take this information into account in order to maximize the chance for patients, particularly women, to benefit from an earliest possible diagnosis of Alzheimer's disease and related disorders.
